# SSVEP-driven BCI authentication with reduced number of EEG electrodes across high and low frequency ranges

**DOI:** 10.3389/fnrgo.2026.1741655

**Published:** 2026-03-26

**Authors:** Ayas Kiser, Atilla Cantürk, Ivan Volosyak

**Affiliations:** 1 BCI Lab Kleve, Faculty of Technology and Bionics, Rhine-Waal University of Applied Sciences, Kleve, Germany; 2 Department of Informatics and Data Science, The Doctoral School for Applied Research in North Rhine-Westphalia (PK NRW), Bochum, Germany

**Keywords:** authentication, brain-computer interfaces (BCIs), electroencephalography (EEG), single stimulus BCI, steady-state visual evoked potentials (SSVEP)

## Abstract

Growing concerns over data privacy, credential theft, and spoofing attacks have highlighted the limitations of traditional authentication methods in high-security settings. To address these challenges, we propose a steady-state visual evoked potential (SSVEP)-based brain-computer interface (BCI) authentication system that verifies short-lived, session-specific identity prompts using neural activity. The proposed system uses a single flickering visual stimulus to encode a unique, system-generated random code that remains unknown to the user. Instead of relying on conscious input, the system directly extracts the user's brain responses to the stimulus. Authentication is achieved by matching the frequency components of the recorded electroencephalography (EEG) signals to those embedded in the visual stimulus, enabling implicit verification without prior training or manual interaction. In an online BCI study involving 21 healthy participants, we evaluated four configurations differing in stimulation frequencies and EEG electrode count. Mean symbol-level accuracy reached 99% (95% CI: 98.3 −99.6) for high-frequency stimulation with three electrodes, 95% (95% CI: 91.1 −98.2) for high-frequency stimulation with a single electrode, 97% (95% CI: 95.1 −98.4) for low-frequency stimulation with three electrodes, and 96% (95% CI: 94.5 −97.8) for low-frequency stimulation with a single electrode. The corresponding mean trial durations were 38.6 s, 76.6 s, 17.2 s, and 27.1 s, respectively. Participants generally rated high-frequency flickering stimuli as more comfortable, whereas setup time and EEG wearability were identified as the main barriers to usability. These findings demonstrate that SSVEP-based authentication can provide accurate and training-free implicit authentication, while also offering potential resistance to spoofing attacks. The results suggest that this passive BCI approach is a promising direction for secure authentication, although practical deployment will require further improvements in speed, comfort, and wearability.

## 1 Introduction

As artificial intelligence, biometric systems, and internet-connected platforms become increasingly integrated into daily services, particularly in banking, healthcare, and identity management, the reliance on digital technologies has grown substantially. As more aspects of daily life move online, including financial transactions and personal data storage, the need to protect sensitive information has become more urgent. Therefore, ensuring robust mechanisms for personal data protection is now a critical priority in the age of widespread digital reliance (Wang et al., 2023; Zhao et al., 2026).

Authentication systems work by comparing a user's current input, such as a fingerprint, facial scan, or brain signal, to previously recorded biometric data. While these systems are sometimes confused with identification, they serve distinct purposes: identification is the act of declaring an identity, while authentication verifies that the claim is legitimate, typically following identification.

Traditional authentication methods can be divided into three categories: knowledge-based (such as passwords), possession-based (such as tokens, credit cards, and key cards), and biometric-based systems. Despite their widespread use, knowledge-based approaches are vulnerable to guessing, theft, or forgetting, and tokens may be lost or stolen (Fidas and Lyras, 2023; Constantinides et al., 2023).

Today, verification and authentication methods that rely on unique biometric features, such as fingerprints or facial recognition, are widely used across many domains. However, these methods face challenges in keeping up with modern technologies due to their vulnerability to data theft and replication attacks (Galbally and Satta, 2016; Zhang et al., 2021).

To enhance security in practical digital services, two-step authentication systems including temporary access codes, commonly referred to as one-time passwords (OTP), have been introduced, particularly for online banking applications. The two-factor authentication method typically involves sending an additional code via SMS or email. In this way, even if malicious actors manage to obtain a user's credentials, they would still be unable to access the system without also having access to the pre-registered email or phone number, thus protecting against unauthorized access.

Online banking, also referred to as internet banking, is a form of digital banking system that enables users to perform a wide range of financial transactions, such as transferring money or remotely accessing and managing their bank accounts, through the internet. Since this system involves access to critical personal financial information, it inherently demands a high level of protection (Wang et al., 2024). In such digital systems where privacy and security are of utmost importance, identity verification is required in order to access the system and perform online banking transactions. The platforms where this verification process takes place are referred to as user authentication systems (Karim et al., 2024).

A BCI enables direct communication between the human brain and external devices, capturing and interpreting neural signals in real time (Wolpaw et al., 2002). Among BCI modalities, EEG-based systems represent a widely used noninvasive approach (Rezeika et al., 2018). These systems have been applied in a range of domains, including assistive technologies, communication tools, and adaptive human-computer interfaces, offering hands-free control (Chen et al., 2015b; Zhang et al., 2021).

Building on these considerations, this study presents an SSVEP-based BCI authentication system tailored to simulate temporary access code-like scenarios using a single flickering stimulus box that sequentially encodes OTP. To evaluate robustness, flexibility, and usability, we tested two frequency sets, low and high, with stimulation frequencies 6.67 Hz, 7.5 Hz, and 8.57 Hz vs. 36 Hz, 40 Hz, and 45 Hz. The code was presented at varying screen positions (left, center, and right) to assess gaze-location robustness, and experiments were conducted using different EEG electrode configurations (three electrodes vs. one electrode) to examine system performance under reduced hardware constraints. The goal was to develop a real-time authentication system that is not only secure and accurate, but also user-friendly, portable, and suitable for future deployment in practical OTP applications.

## 2 Related work

In an effort to address the limitations of conventional biometric systems, numerous studies have explored the use of brain signals for user authentication and identification purposes (Wang et al., 2020; Behrouzi and Hatzinakos, 2022; Zhao et al., 2026). Many of these works initially focus on simplifying the system by leveraging the user's mental states, such as eyes-closed resting or eyes-open resting conditions. This approach is based on the idea of acquiring neural data in a natural and effortless manner, as it does not require the user to perform any specific cognitive task (Jalaly Bidgoly et al., 2020). This distinction is further emphasized by Gui et al. (2019), who systematically categorize brain biometric protocols into resting-state and task-based paradigms. In resting-state protocols, EEG is acquired while users remain at rest with their eyes open or closed, without performing any explicit cognitive task. Owing to their simplicity and ease of continuous data collection, such protocols have been widely adopted in brain biometric systems for both authentication and identification purposes. However, the authors also note that the absence of explicit task constraints may limit control over the underlying brain states, motivating the exploration of more structured, stimulus-driven protocols in subsequent studies.

Gui et al. (2019) note that many brain biometric systems rely on event-related potentials (ERPs) rather than raw EEG signals, as ERPs represent neural responses that are time-locked to specific stimuli and therefore provide higher specificity and improved signal-to-noise ratio. Visual evoked potentials (VEPs) constitute a particular class of ERPs elicited by visual stimulation and have also been explored for biometric applications. However, because VEPs predominantly reflect activity in the visual cortex, their effective use requires carefully controlled experimental designs to minimize interference from unrelated cognitive, semantic, or auditory processes. While ERP- and VEP-based protocols offer advantages in terms of stimulus specificity and robustness, they also impose stricter constraints on stimulus presentation compared to unconstrained EEG acquisition, highlighting the importance of well-designed visual paradigms for practical biometric systems.

Another frequently used visual paradigm, SSVEP, refers to brain responses elicited by repetitive visual stimuli flickering at constant frequencies, typically involving periodic components that match the stimulation frequency and its harmonics (Regan, 1966). SSVEPs offer a practical pathway for high-throughput communication in BCI applications (Chen et al., 2023). Moreover, the robustness and lack of required training make SSVEP-based systems well-suited for real-time control scenarios and user-friendly interaction (Liu et al., 2022).

SSVEP signals have been comparatively less explored for person identification than for conventional BCI control tasks. Early SSVEP-based biometric studies typically involved a small number of participants and stimulation frequencies, relying on hand-crafted temporal or spectral features combined with classical classifiers such as kNN or distance-based similarity measures. Oikonomou (2023) highlighted these limitations and demonstrated that subject-specific spatial patterns in multichannel SSVEP recordings can be effectively exploited using deep learning and spatial filtering approaches. This work emphasized the importance of spatial information and larger stimulus sets for achieving high discriminability in SSVEP-based biometric systems.

Further extending this line of research, Oikonomou (2025) proposed a sparse representation classification (SRC) framework for SSVEP-based person identification and verification. The study emphasized that existing SSVEP biometric systems often rely on small datasets, a limited number of stimulation frequencies, and hand-crafted temporal or spectral features combined with classical or deep learning classifiers. To address these limitations, the proposed framework integrates spatial filtering through Filter-Bank Common Spatial Patterns (FBCSP) with SRC, exploiting multichannel SSVEP recordings to construct discriminative and overcomplete dictionaries. By incorporating spatial patterns and sparse representations, the approach demonstrated improved robustness under noisy and short-duration EEG conditions, at the cost of increased system complexity and reliance on training data.

For another instance, in a previous study from our laboratory (Ngan Le et al., 2025), a BCI system was integrated with a simulation of an online banking platform for authentication purposes. The core idea of the study was to replace manual entry of the OTP with a code-modulated visual evoked potential (cVEP)-based visual paradigm displayed on a monitor. In this setup, each digit was assigned a specific m-sequence and its circularly left-shifted variants. Users were asked to fixate their gaze on the stimuli corresponding to each digit of the code they saw on the screen. As a result, the study reported an average classification accuracy of 69.70% and a mean trial completion time of 45.04 seconds.

Taken together, existing studies highlight complementary strengths and limitations in EEG-based biometric systems. Resting-state approaches offer simplicity but limited control over brain states, while ERP- and VEP-based paradigms provide stimulus specificity at the cost of increased experimental complexity (Gui et al., 2019). Recent SSVEP-based biometric studies have demonstrated that rich spatial information and learning-based frameworks can substantially improve discriminability (Oikonomou, 2023, 2025), at the cost of relying on multichannel hardware, training procedures, and increased computational demands. In parallel, prior work from our laboratory explored an OTP-like authentication scenario using a cVEP-based visual paradigm (Ngan Le et al., 2025), demonstrating the feasibility of brain-based authentication in realistic application contexts, but with moderate accuracy and relatively long completion times. Motivated by these observations, the present study aims to combine the practical advantages of SSVEP stimulation with a simplified, low-channel hardware configuration and a training-free FBCCA framework, investigating whether an OTP-style authentication process can be achieved with higher accuracy and shorter completion time under more deployable system constraints.

## 3 Material and methods

This section describes the hardware setup, stimulus presentation, experimental design, and provides details on the classification methods and the authentication interface for short-lived code entry. The dataset used in this study was collected during an online EEG-based BCI experiment conducted with 21 healthy participants. Participants were recruited remotely and completed the experiment using a standardized experimental protocol under controlled stimulus presentation conditions. Demographic information, including age, gender, country of residence, vision correction, and handedness, was recorded for each participant. All participants provided informed consent prior to participation. An overview of participant demographics and the hardware configuration used for data acquisition is provided in the following tables.

### 3.1 Participants

The study involved 21 healthy volunteers (mean age = 26.33 ± 4.77 years), comprising 12 females (57.14 %) and 9 males (42.86 %). The female group consisted of participants who identified themselves as female on the questionnaire, while the male group comprised those identifying as male. Participants were recruited on a first-come, first-served basis. An open call was published via the university's internal system to invite individuals interested in participating in experiments. No pre-selection or screening was conducted during the registration phase. Eligibility was determined on-site based on participants' responses to an informed consent form, which outlined specific exclusion criteria. These included a history of epilepsy or other neurological, psychiatric, or psychological conditions; known allergies to electrode gel or similar substances; and current influence of alcohol or drugs.

As summarized in Table 1, both groups represented a broad demographic distribution, with the female group spent most of the time of their lives in eight different countries and the male group eight, amounting to thirteen different countries overall, supporting the study's diversity. In terms of visual correction, 11 participants indicated that they did not require any form of assistance. Among the 10 participants who typically used visual correction, one reported not using it during the experimental sessions (subject 2). The BCI study was non-invasive and did not cause any pain. This study was approved by the ethical committee of the medical faculty at the University Duisburg-Essen (24-11957-BO). Participants were fully informed about possible risks and the experimental procedures prior to participation. All participants provided written consent in line with the Declaration of Helsinki and participant data were stored anonymously. Volunteers were compensated with 10€ and retained the right to withdraw from the study at any time without providing justification.

**Table 1:** Demographic distribution of participants.

**Participant**	**Age**	**Gender**	**Primary country of residence**	**Vision correction**	**Dominant hand**
1	20	M	Germany	Yes	Right
2	21	F	Peru	Yes*	Right
3	23	M	Colombia	No	Right
4	39	F	Mexico	No	Right
5	23	F	India	Yes	Right
6	28	F	Germany	Yes	Right
7	25	F	Turkey	No	Right
8	23	F	India	No	Right
9	35	M	Tanzania	Yes	Right
10	23	F	Brazil	Yes	Right
11	23	M	India	No	Right
12	31	F	Mexico	Yes	Left
13	25	F	Germany	No	Right
14	24	M	India	No	Right
15	27	M	Uganda	No	Right
16	33	F	Iran	No	Right
17	24	F	Netherlands	Yes	Right
18	25	M	Nigeria	No	Ambidextrous
19	27	M	Hungary	Yes	Right
20	25	M	Turkey	No	Right
21	29	F	Turkey	Yes	Right

“Yes*” indicates participants who required vision correction but did not use it during the experiment.

### 3.2 Hardware

The experimental setup included standard EEG acquisition hardware and signal processing components. A summary of the hardware and processing environment, including EEG amplifier, EEG electrodes, filters, and computing infrastructure, is provided in Table 2.

**Table 2:** Hardware used in the BCI experiment.

**EEG signal acquisition**	
Amplifier	g.USBamp (g.tec, Schiedlberg, Austria)
Sampling freq. (*F*_*S*_)	600 Hz blockwise, 30 samples/block
Channels	3 passive Ag/AgCl electrodes (*O*_1_, *O*_*Z*_, *O*_2_)
Gel	Abrasive electrode gel (impedances < 5 kΩ)
Locations (3-channel)	*O*_1_, *O*_*Z*_, *O*_2_
Location (1-channel)	*O*_*Z*_
Ground | Reference	*AF*_*Z*_ |*C*_*Z*_
**Digital filters (g.USBamp)**	
Notch | Band-pass	48–52 Hz | 2–60 Hz
**Signal processing equipment**	
PC	Dell precision desktop
CPU	Intel Core i9-10900K (up to 5.3 GHz)
RAM	16 GB RAM
Graphics	NVIDIA RTX-3070
OS	Windows 10 education (21H2, 64-bit)
Display	Asus ROG swift PG258Q (1,920 × 1,080 @360 Hz)

### 3.3 Experimental protocol

**Figure 1:**
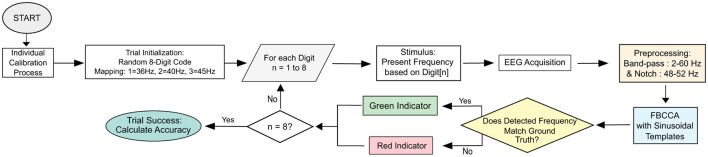
Flowchart of the proposed real-time SSVEP-based authentication system. The pipeline shows random code generation, frequency-coded visual stimulation, EEG acquisition, minimal preprocessing, FBCCA-based signal processing, and feedback-controlled digit-by-digit authentication.

The experimental protocol was designed to evaluate the real-time performance of the sequential 8-digit authentication process. The overall procedural logic, ranging from initial participant preparation to the final trial-based feedback generation, is systematically illustrated in the flowchart in Figure 1. This pipeline ensures that each participant undergoes a standardized sequence of calibration and multi-configuration testing, with the core signal processing loop handling the digit-by-digit verification in real time.

#### 3.3.1 Preparation and participant briefing

All experimental sessions were conducted at the BCI-Lab of Rhine-Waal University of Applied Sciences (HSRW). Upon arrival, participants were first presented with a written consent form outlining the purpose, procedures, and voluntary nature of the study. Only participants who did not meet the exclusion criteria (described in Section 3.1) proceeded with the experiment.

After signing the consent form, participants received a detailed information sheet and were given sufficient time to read it. They then completed a demographic form and a pre-experiment questionnaire via an online system. During the EEG setup, participants were briefed in detail about the experiment and were encouraged to ask any relevant questions. The procedure was explained again verbally, and any remaining questions were addressed before the beginning of the main experiment.

#### 3.3.2 Experimental configurations and counterbalancing

The experiment was carried out under four configurations that differed in electrode count and frequency range: a three-electrode high-frequency setup (visual stimuli at 36, 40, and 45 Hz), a three-electrode low-frequency setup (6.67, 7.50, and 8.57 Hz), a single-electrode high-frequency setup (36, 40, and 45 Hz), and a single-electrode low-frequency setup (6.67, 7.50, and 8.57 Hz).

To control for possible order effects, participants were divided into two counterbalanced groups. Odd-numbered participants began with the high-frequency configurations, whereas even-numbered participants started with the low-frequency configurations. In all cases, sessions began with the three-electrode setup, followed by the corresponding single-electrode setup using the same frequency order (high–low or low–high).

#### 3.3.3 Visual stimulation task

The experimental task employed an SSVEP-based visual stimulation setup consisting of three boxes displayed horizontally on the screen. At the beginning of each trial, one of the boxes (left, center, or right) was randomly selected and highlighted with a green border, designating it as the target stimulus. Participants were instructed to fixate their gaze solely on the highlighted box until the end of the trial.

An overview of the complete authentication pipeline, including stimulus presentation, EEG processing, classification, and feedback generation, is illustrated in Figure 1.

**Figure 2:**
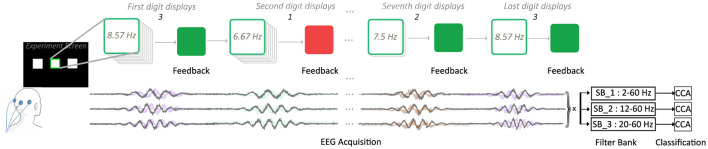
Signal processing workflow of a sequential SSVEP-based authentication trial. The diagram illustrates how an 8-digit OTP (composed of digits 1, 2, and 3) is processed through a single flickering stimulus over time. The multiple stimulus boxes shown represent the discrete temporal steps of the sequence (e.g., Digit 3 at *t*_1_, Digit 1 at *t*_2_, ..., Digit 2 at *t*_7_, and Digit 3 at *t*_8_), rather than concurrent targets. For each step, the system performs real-time preprocessing and FBCCA classification by correlating the EEG data (*X*) with target-specific sinusoidal reference signals (*Y*). Immediate visual feedback is provided for each digit before the stimulus automatically transitions to the next frequency in the sequence, ensuring a seamless 8-digit authentication flow.

For each configuration, participants completed four authentication trials. Each trial corresponded to an 8-digit code randomly generated by the system. Each digit was encoded by a distinct flickering frequency, and the entire sequence of eight digits was visually presented within the same stimulus box. Following each classification, the system provides immediate visual feedback: when the system correctly recognized a digit, the stimulus box briefly expanded and turned green to indicate positive feedback. For incorrect classifications, the box expanded in red instead. Short feedback intervals separated consecutive digits while preserving the overall continuity of the flickering sequence. Participants were instructed to maintain fixation on the designated box without making any manual responses or selections. This digit-wise feedback allowed participants to observe system performance in real time, as illustrated in Figure 2, while the overall authentication process was finalized only after all eight digits had been presented.

#### 3.3.4 Calibration procedure

Before the main experimental trials, an individual calibration phase was conducted for each participant to maximize classification accuracy. Two parameters were adjusted: the *classification threshold* and the *minimum EEG signal window*. The classification threshold, determining the confidence level required to accept a decision, was gradually tuned within the range of 0.2 to 0.5 in small increments while participants fixated on the stimulus. If reliable classification could not be achieved at a given threshold, the minimum signal window, representing the shortest segment of EEG data usable for classification, was incrementally extended until optimal accuracy was reached.

If perfect (100%) accuracy could not be achieved, calibration concluded once the highest attainable accuracy was reached within the available session time, and the corresponding threshold and window parameters were retained for subsequent trials. Calibration was performed separately for high- and low-frequency conditions to account for frequency-dependent variations in SSVEP amplitudes. The finalized parameter values for each participant were then kept constant during the main experiment to ensure comparability across sessions.

#### 3.3.5 Post-experiment phase

After completing all trials under every configuration, participants filled out post-experiment questionnaires. The session concluded with assistance in removing the EEG cap.

### 3.4 Stimulus presentation

In this study, the visual stimulus paradigm consisted of three horizontally aligned square boxes that flickered continuously at predetermined frequencies throughout each trial (see Figure 2). During every trial, one of the three boxes was randomly selected as the target and highlighted with a green border to guide the participant's attention. The target could appear on the left, center, or right side of the screen. More importantly, even though only one box was designated as the target, the two non-target boxes continued flickering at the other two frequencies from the three-frequency set. This intentional design ensured that classification depended solely on the participant's focus on the highlighted target. At the same time, it introduced a realistic challenge: an accidental and sufficiently long gaze at a non-target could lead to the detection of an incorrect digit.

Additionally, this randomization of the target box position (left, center, or right) was intended to assess whether system performance remained robust across varying gaze directions. Each trial involved the system internally generating a unique and random 8-digit authentication code composed of digits 1, 2, and 3 (e.g., 22313132). Each digit was mapped to a specific flickering frequency, either from a low-frequency set (6.67 Hz, 7.50 Hz, or 8.57 Hz) or high-frequency set (36 Hz, 40 Hz, or 45 Hz), respectively, depending on the experimental setup. These frequencies were assigned to represent digit values (e.g., digit one was coded as 6.67 Hz or 36 Hz, depending on the current frequency set, low vs. high), consistently throughout the experiment.

The stimulation frequencies were selected as integer divisors of the 360 Hz monitor refresh rate, ensuring precise frame-locked flickering without timing artifacts, see (Volosyak et al., 2009) for more details. The frequencies (6.67, 7.50, and 8.57 Hz) were chosen as they fall within the range known to elicit strong SSVEP responses (Herrmann, 2001; Wang et al., 2006), while avoiding the 10-15 Hz range due to overlap with endogenous alpha rhythms during eyes-closed states, which can cause false positives (Herrmann, 2001; Vialatte et al., 2010). Frequencies of a high-frequency set (36, 40, and 45 Hz) were selected to minimize visual discomfort while maintaining technical stability (Vialatte et al., 2010; Volosyak et al., 2011; Diez et al., 2011). In particular, 40 Hz has been frequently reported as a robust stimulation frequency within the gamma range (Regan, 1989). The randomly generated code was represented by its corresponding frequencies, with each digit presented for at least the minimum time window and remaining active until a classification was made. In cases of incorrect classification, the trial continued automatically to the next digit after feedback was displayed, ensuring consistent trial duration and uninterrupted sequence flow. As a result, the box appeared to flicker continuously, but the underlying frequency changed in discrete steps according to the sequence of digits. This design allowed for the presentation of an entire 8-digit code through a single stimulus without requiring user interaction or switching between different stimuli.

### 3.5 Classification

This study employed the Filter Bank Canonical Correlation Analysis (FBCCA) technique as described in Chen et al. (2015a) to enhance user-friendliness and minimize the need for extensive calibration. FBCCA, an extension of canonical correlation analysis (CCA), improves frequency detection in SSVEP-based BCI systems by leveraging both the fundamental and harmonic components of neural responses. Unlike machine-learning-based classifiers, FBCCA requires no subject-specific training phase, thereby reducing calibration effort and improving accessibility for end-users.

In this framework, EEG signals are first decomposed into multiple frequency bands using a filter bank, after which CCA is performed between each band-pass-filtered EEG component and a set of sine-cosine reference templates corresponding to the stimulation frequencies and their harmonics. The frequency yielding the highest combined correlation across all sub-bands is identified as the attended target. Following Chen et al. (2015a), we applied multiple sub-bands to capture both fundamental and harmonic SSVEP components. Specifically, for the low-frequency set (6.67, 7.50, and 8.57 Hz), the EEG signals were filtered into several sub-bands. For low frequency setups consisted of 3 sub-bands 2–60, 12–60, and 20–60 Hz. For the high-frequency set (36, 40, and 45 Hz), a single broad band of 35–60 Hz was used to encompass the relevant frequencies.

To further enhance signal-to-noise ratio, the EEG data were separated into multiple sub-bands using a filter bank composed of five fourth-order Butterworth bandpass filters, each defined by distinct lower and upper cutoff frequencies.

The upper cutoff of 60 Hz was selected in accordance with the amplifier's predefined bandpass filters available. Applying sub-band filtering isolates frequency-specific SSVEP responses, thereby improving classification accuracy. For each stimulus frequency *f*_*kl*_∈{6.67, 7.50, 8.57} Hz and *f*_*kh*_∈{36, 40, 45} Hz, reference signals were generated using sine and cosine functions at both the base frequency and its harmonics. The sampling frequency was *f*_*s*_ = 600Hz, with *H* = 4 harmonics for low frequency set and *H* = 1 harmonic for high frequency set and *N* samples indexed as *n* = 0, 1, …, *N*−1.

The FBCCA algorithm was applied to both low- and high-frequency conditions. The only differences were the number of filter banks used and the number of harmonics included in the reference signal set. Since only a single filter band and the base frequencies (with one harmonic) were used for high-frequency stimuli, the algorithm in this case essentially behaves like standard CCA, but with the EEG signals pre-filtered by a bandpass filter.

The reference matrix **Y**_*k*_(*t*) for each frequency *f*_*k*_ was defined as:


$$\begin{array}{l} {\text{Y}}_{k}\left(t\right)=\left[\begin{array}{c} \sin\left(2\pi f_{k}\frac{n}{f_{s}}\right)\\ \cos\left(2\pi f_{k}\frac{n}{f_{s}}\right)\\ \sin\left(2\pi \cdot 2f_{k}\frac{n}{f_{s}}\right)\\ \cos\left(2\pi \cdot 2f_{k}\frac{n}{f_{s}}\right)\\ \vdots \\ \sin\left(2\pi \cdot Hf_{k}\frac{n}{f_{s}}\right)\\ \cos\left(2\pi \cdot Hf_{k}\frac{n}{f_{s}}\right) \end{array}\right]\;\text{for }n=0,1,\ldots ,N-1 \end{array}$$ (1)

As illustrated in Figure 2, the classification process analyzed EEG responses elicited while participants fixated on the target stimulus. Each flickering frequency, corresponding to a specific digit, resulted in a distinct SSVEP pattern in the acquired EEG. These signals were processed in real time using FBCCA, correlating the filtered EEG components with frequency-specific reference templates to determine the best-matching stimulus frequency. After each classification, immediate visual feedback was displayed, and the process continued until all eight digits of the code were decoded sequentially from the participant's brain responses.

Beneath the stimulus timeline, the flicker frequencies are depicted as color-coded square waveforms with corresponding black–white blocks symbolizing the visual flicker. Below these, schematic EEG waveforms illustrate frequency-specific SSVEP responses. The recorded signals (**X**) are decomposed into multiple sub-bands using the filter bank, and CCA is performed between each sub-band signal and the reference templates (**Y**). The resulting correlation coefficients are combined across sub-bands to enhance robustness against noise and harmonic overlap, following the principles of FBCCA (Chen et al., 2015a). The final classification result produces real-time visual feedback, completing the loop of the authentication trial. For the low-frequency setup (6.67, 7.50, and 8.57 Hz), three sub-bands were defined (*SB*_1_: 2–60 Hz, *SB*_2_: 12–60 Hz, and *SB*_3_: 20–60 Hz) to specifically target the fundamental frequency and the subsequent 2nd and 3rd harmonics while progressively suppressing low-frequency background EEG activity. For the high-frequency setup (36, 40, and 45 Hz), a single broad band of 35–60 Hz was utilized. This decision was based on the fact that the harmonics for these frequencies (starting at 72 Hz) exceeded the 60 Hz hardware bandpass limit of the amplifier. All FBCCA-related parameters used for the low- and high-frequency stimulation conditions are summarized in Table 3. The table provides an overview of the selected stimulus frequencies, number of sub-bands, sub-band ranges, number of harmonics included in the reference signals, and the weighting coefficients applied during sub-band fusion. The complete set of FBCCA parameters for both stimulation frequency ranges is summarized in Table 3. These parameters were selected following the recommendations of Chen et al. (2015a) to balance classification accuracy and real-time performance under reduced-channel constraints.

**Table 3:** FBCCA parameters for low-frequency and high-frequency setups.

**Parameter**	**Low-frequency setup**	**High-frequency setup**
Stimulus frequencies	6.67, 7.50, 8.57 Hz	36, 40, 45 Hz
Number of sub-bands (*N*)	3	1
Sub-band ranges (Hz)	2–60, 12–60, 20–60	35–60
Reference harmonics (*H*)	4	1
Weighting coefficients	*a* = 1.25, *b* = 0.25	N/A

#### 3.5.1 Target identification using FBCCA

For each stimulus frequency and sub-band, we first compute the canonical correlation coefficients and then combine them into a single FBCCA score via a weighted sum. Lower-index (lower-frequency) sub-bands receive greater emphasis because they typically retain more reliable SSVEP content. We adopt a non-linear decay for the weights, approximated by $w_{i}={\left(i+1\right)}^{-1.25}+0.25$, where *i* denotes the sub-band index. A candidate frequency *f*_*k*_ is accepted as the intended command only when the correlation value of the highest-scoring class exceeds that of the second-highest class by at least a predefined threshold, and the timing criteria are met.

The classifier runs on a sliding window: the EEG buffer is updated continuously and correlations are recomputed every 50 ms (30 samples), yielding a 20 Hz update rate. A decision is issued only if:

- At least 300 samples (unless it was increased by the experimenter) have elapsed since stimulus onset
- The highest FBCCA score exceeds the runner-up by at least the threshold value decided by the experimenter (0.30 by default)

When the buffer reaches its maximum span (1,800 samples), older samples are dropped as new ones arrive, maintaining a fixed window.

Once these criteria are satisfied, the system executes the classification routine, clears the EEG buffers, and triggers a 0.5 s gaze-shift period before starting the next cycle.

Each decision selected one of three target frequencies mapped to digits: 6.67 Hz → 1, 7.5 Hz → 2, and 8.57 Hz → 3 for low frequency set and 36 Hz → 1, 40 Hz → 2, and 45 Hz → 3 for high frequency set.

The above pipeline was implemented in C++ using Microsoft Visual Studio Professional 2022 with the ISO C++17 standard. The application handled (i) EEG acquisition from the g.USBamp and (ii) real-time classification.

### 3.6 Evaluation measures of BCI performance

System performance was evaluated using two primary measures. **Accuracy** (*P*) was defined as the proportion of correctly classified digits relative to the total digits classified in a trial. **Completion time** (*s*) was defined as the elapsed time needed to complete one trial, from the onset of the first stimulus flicker to the classification of the final digit. For those interested in calculating or analyzing classification accuracy in related BCI experiments, we provide access to our group's publicly available calculator, see https://bci-lab.hochschule-rhein-waal.de/en/itr.html.

### 3.7 Questionnaires

**Figure 3:**
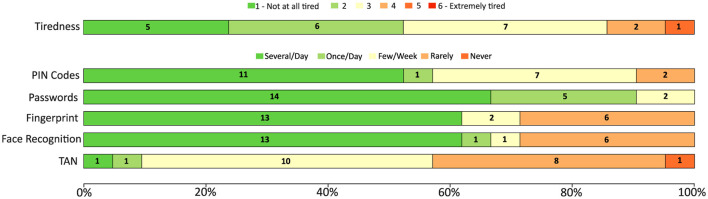
Pre-questionnaire results summarizing participants' self-reported tiredness levels and frequency of use of common authentication methods. The stacked bar chart shows normalized response distributions (percentage of participants), with numerical labels indicating the number of participants per response category. Tiredness was rated on a six-point Likert scale ranging from 1 (not at all tired) to 6 (extremely tired). Usage frequency was reported for PIN codes, passwords, fingerprint, face recognition, and TAN, with response options ranging from “several times per day” to “never.” Results are shown for all participants prior to the experimental session.

To complement the objective performance metrics collected during the experiment, participants were asked to complete a two-stage questionnaire, consisting of pre- and post-experiment sections. Each participant was assigned a unique subject number and an automatically generated anonymous identifier (ID) produced by a dedicated software developed in our lab. This mechanism was employed solely to enable anonymous and consistent matching of questionnaire data with experimental outcomes, ensuring participant privacy while maintaining data integrity throughout the analysis. The pre-questionnaire included demographic and contextual variables known to influence BCI performance (see Table 1). These included age, which has been shown to correlate with neural responsiveness; country of residence, providing insight into cultural and demographic diversity; gender, as EEG-based studies have reported gender-related differences; and dominant hand, given its relation to hemispheric specialization. Participants were also asked to report their level of fatigue prior to the session (as shown in Figure 3), the number of hours they had slept the night before, and whether they used any visual aids. To control for potential confounding effects on neural activity and alertness, participants were asked whether they had consumed substances such as caffeine, alcohol, painkillers, antibiotics, or sleep aids within the last 12 h. Sensitive items were accompanied by short justifications to increase transparency. The fatigue question was repeated in the post-experiment questionnaire to assess the cognitive load induced by the session and to gather user feedback for future refinements.

Following the experiment, participants completed a post-questionnaire designed to capture subjective feedback regarding their user experience and perceived system usability. As a follow-up to the pre-experiment fatigue measure, participants were again asked to rate their current level of tiredness on the same 6-point scale to assess the cognitive load imposed by the system. Additional questions addressed visual comfort, asking whether participants experienced eye discomfort and to what extent they noticed the flickering stimuli. Participants were also asked to evaluate the mental effort required by the task, again on a 6-point scale. To explore the usability of different stimulus frequencies, they were asked which flickering frequency (high or low) was more comfortable and more visible for them, and which they would prefer if required to use the system in a real-world setting.

Participants were further invited to consider the broader applicability of the system, including whether they would consider using a BCI-based authentication method instead of traditional approaches. An open-ended question was included to collect suggestions for system improvement based on their personal experience. Finally, a multiple-choice question allowed participants to indicate any concerns or limitations, such as comfort, speed, accuracy, or privacy, that might prevent them from adopting such a system in daily life. These subjective insights were intended to guide future iterations of the system toward more user-friendly and accessible applications.

## 4 Results

This section presents a comprehensive analysis of the experimental results, focusing on four key dimensions that impact system performance. First, the effect of electrode configuration is evaluated by comparing three-electrodes and one-electrode setups. Second, stimulus frequency type is examined by analyzing the differences between low and high-frequency conditions. Third, performance is assessed based on the index of each digit within the one-time code sequence to investigate potential fatigue or latency across the trial. Lastly, the completion time and classification accuracy are compared among individual stimulus frequencies within each frequency group, offering insights into frequency-specific performance variations.

As shown in Table 4, clear performance differences were observed across the four experimental conditions. The highest mean accuracy was achieved in the three-electrodes high-frequency condition (99% ± 3.5), while the lowest was found in the one-electrode high-frequency condition (95% ± 9.95). In terms of completion times, the shortest average was obtained in the three-electrodes low-frequency condition (17.2 s ± 9.7), whereas the longest time occurred in the one-electrode high-frequency setup (76.6 s ± 102.1). On the other hand, the lowest accuracies obtained across participants for each condition are as follows: three-electrodes high-frequency 97%, three-electrode low-frequency 88%, one-electrode high-frequency 75% and one-electrode low-frequency 91%. These findings indicate that increasing the number of electrodes generally provided higher accuracies, while single-electrode conditions, particularly with high-frequency stimuli, showed greater variability across participants. Comparing frequency conditions, lower frequencies tended to yield shorter and more stable completion times, whereas three-electrodes high frequency set-up resulted in higher accuracies overall, pointing toward a trade-off between speed and precision. The 95% confidence intervals (CI) reported in Table 4 further support these observations by quantifying the reliability of the estimated mean performance across subjects. In particular, narrower confidence intervals were observed for three-electrode configurations, indicating more consistent accuracy and completion time estimates across participants. In contrast, the wider confidence intervals associated with the single-electrode high-frequency condition reflect increased inter-subject variability, highlighting the sensitivity of reduced-channel setups to individual factors.

**Table 4:** Averaged classification accuracy (%) and task completion time(s) for each subject using two electrode configurations (3-electrode and 1-electrode) and two stimulation frequency ranges: Low Frequency (LF) and High Frequency (HF).

	**3-electrode**				**1-electrode**			
**Subj. No**.	**LF**		**HF**		**LF**		**HF**	
	**Acc (%)**	**Time (s)**	**Acc (%)**	**Time (s)**	**Acc (%)**	**Time (s)**	**Acc (%)**	**Time (s)**
1	94	25,64	100	12,74	97	16,69	100	56,4
2	97	16,99	100	27,1	91	72,25	75	62,08
3	100	12,44	100	42,16	97	16,58	91	113,3
4	100	16,39	100	15,23	100	27,09	100	48,36
5	91	28,39	97	125,75	100	22,69	97	48,78
6	100	15	97	20,59	94	40,56	100	76,21
7	97	28,18	97	95,75	100	34,14	94	79,13
8	94	44,29	100	65,11	94	73,65	100	64,83
9	97	28,68	97	51,45	100	27,98	81	52,14
10	94	10,09	100	15,66	91	36,93	100	10,61
11	100	14,3	100	50,66	94	37	78	246,66
12	97	8,25	97	10,78	97	8,65	97	32,83
13	100	9,54	100	11,69	100	9,2	100	36,46
14	94	9,41	100	32,16	97	20,65	94	374,06
15	97	10,26	97	25,59	100	13,16	100	14,33
16	88	23,29	100	63,98	94	14,16	97	55,68
17	100	11,9	100	8,39	100	18,5	100	7,93
18	100	18,1	97	104,9	100	43,14	88	188,95
19	94	11,83	100	15,55	91	13,64	100	20,3
20	100	8,2	100	7,81	91	11,65	97	10,26
21	100	9,78	100	7,88	94	11,28	100	9,83
Mean ± SD	97 ± 6,8	17,19 ± 9,73	99 ± 3.5	38,61 ± 42,55	96 ± 7,0	27,12 ± 21,66	95 ± 9,95	76,62 ± 102,14
**95% CI**	[95,1, 98,4]	[12,9, 21,4]	[98,3, 99,6]	[22,8, 54,4]	[94,5, 97,8]	[18,6, 35,6]	[91,1, 98,2]	[35,5, 117,7]

Each pair of columns reports accuracy and time per subject, showing how electrode reduction and stimulation frequency influence performance. The last two rows present the overall mean with standard deviation and the corresponding 95% confidence intervals (CI) across all subjects.

The lowest overall classification accuracy was observed in the high-frequency, single-electrode configuration, where one participant (Subject 2) achieved 75%. Questionnaire data revealed that this participant had one of the least sleep duration among all participants on the night prior to the experiment and was also the only one with a vision correction prescription who did not use it during the session. These factors likely contributed to reduced visual focus and overall SSVEP signal quality, explaining the lower performance. Notably, some participants exhibited extremely long trial durations in the one-electrode high-frequency condition, which substantially increased variance. Nevertheless, the consistently high accuracies across all conditions underline the reliability of the proposed system. Furthermore, these results align with earlier findings showing that completion times did not systematically depend on the digit position within the code, suggesting that trial performance was not influenced by sequential fatigue effects.

**Figure 4:**
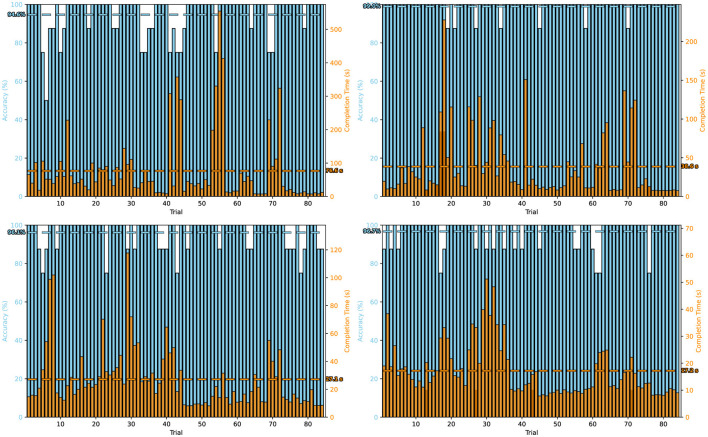
Trial-wise accuracy and completion time across all experimental setups. Panels represent: High Frequency-1 electrode **(top left)**, High Frequency-3 electrodes **(top right)**, Low Frequency-1 electrode **(bottom left)**, and Low Frequency-3 electrodes **(bottom right)**.

Figure 4 presents trial-wise accuracy and completion time values for each participant across the four configurations. The panels are organized as follows: top left shows high-frequency with one electrode, top right shows high-frequency with three electrodes, bottom left shows low-frequency with one electrode, and bottom right shows low-frequency with three electrodes. This visualization highlights both variability across individual trials and the overall stability of each condition. Notably, the high-frequency three-electrode configuration achieved near-perfect accuracy in almost all trials, while single-electrode setups exhibited larger variability. In particular, high-frequency single-electrode trials showed several instances of extended completion times, resulting in higher variance. In contrast, the low-frequency conditions demonstrated more balanced completion times but slightly reduced accuracy compared to high-frequency setups. These findings further confirm that system performance is sensitive to both electrode configuration and frequency range.

### 4.1 Performance analysis based on number of used EEG electrodes

When comparing the effect of electrode number, the three-electrode setup consistently outperformed the single-electrode configuration. On average, accuracies were higher and more stable with three electrodes (LF: 97 %, HF: 99 %) compared to one electrode (LF: 96 %, HF: 95 %). Completion times also showed less variability in the three-electrode condition, particularly in the low-frequency setup, which achieved the fastest average performance (17.2 s). In contrast, the one-electrode high-frequency condition produced both the lowest mean accuracy and the highest variability in completion times, with some participants requiring more than 200 s to complete a trial.

**Figure 5:**
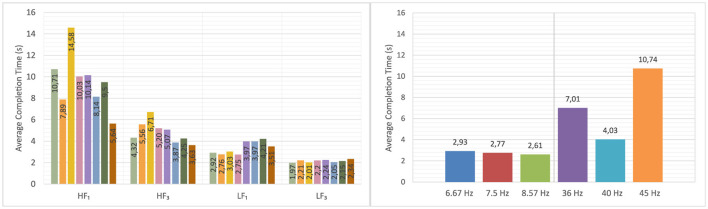
**Left**: Average completion time per digit position within an 8-digit trial, shown for all four experimental configurations (high-frequency with one electrode, high-frequency with three electrodes, low-frequency with one electrode, and low-frequency with three electrodes). **Right**: Average completion times across the six individual stimulus frequencies (36, 40, and 45 Hz for high-frequency; 6.67, 7.5, and 8.57 Hz for low-frequency).

Figure 5 presents two aspects of trial performance based on completion time. On the left, average completion times are shown for each digit position (from 1st to 8th) within the authentication code. The four conditions, high-frequency with one electrode, high-frequency with three electrodes, low-frequency with one electrode, and low-frequency with three electrodes, are compared to evaluate whether visual fatigue or attention loss over time affects participant performance. Results did not reveal a systematic increase or decrease across digit positions, suggesting that performance was not strongly affected by trial progression.

On the right, the figure compares average completion times for each individual stimulus frequency used in the experiment. While some variability was observed across frequencies, no single frequency consistently outperformed the others across all conditions. Within the high-frequency set, the 40 Hz stimulus yielded the shortest average completion times, suggesting a practical trade-off between performance and comfort. Across conditions, the ordering of fastest and slowest positions showed no monotonic trend, indicating that within-trial fatigue was limited. Low-frequency conditions produced shorter per-digit times overall, consistent with faster lock-in at lower flicker rates.

### 4.2 Effect of stimulus frequency on performance

Our results demonstrated clear differences between low- and high-frequency stimulation. The three-electrode high-frequency configuration achieved the highest overall accuracy (99 %) with relatively short completion times, indicating that high-frequency stimuli can provide efficient performance when sufficient spatial information is available. Conversely, the one-electrode high-frequency setup produced the lowest average accuracy (95 %) and the longest completion times, highlighting the sensitivity of high-frequency SSVEP paradigms to reduced electrode coverage.

Low-frequency conditions yielded more balanced completion times across participants, although accuracies were lower than in the high-frequency three-electrode setup. Overall, low-frequency conditions produced shorter completion times but slightly lower accuracy. These patterns indicate a performance trade-off between speed and precision depending on the stimulation frequency.

### 4.3 Gender-based analysis of performance

We examined performance differences by gender. Female participants achieved a mean classification accuracy of 97%, while male participants reached 96%. In terms of completion time, females averaged 33.16 s per trial compared to 48.85 s for males. Although the accuracy difference was small, female participants generally completed trials more quickly than males.

### 4.4 Evaluation of questionnaires

**Figure 6:**
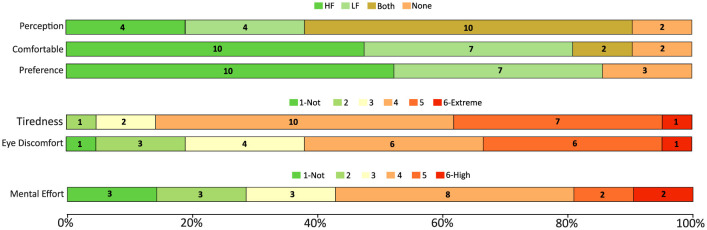
Summary of post-questionnaire results covering system usability (mental effort, eye discomfort, and tiredness) and perception, comfort, and preference regarding high- vs. low-frequency flicker.

Figure 6 summarizes the questionnaire outcomes across two categories. The bottom section shows participant ratings of tiredness (after the session), eye discomfort, and mental effort on a 6-point likert scale (1 = very low, 6 = very high). The middle section illustrates the frequency of use for common authentication methods (TAN, face recognition, fingerprint, passwords, PIN codes), ranging from “several times a day” to “never.” The top section contrasts high-frequency (HF) and low-frequency (LF) flicker by asking participants whether the flicker was perceptible, which frequency felt more comfortable, and which they would prefer, with options including HF, LF, both equally, or no preference/uncomfortable.

The post-session questionnaire provided insights into user comfort, preferences, and the perceived practicality of the proposed system. As shown in Figure 6, the majority of participants were able to clearly perceive both high- and low-frequency flickers, with a slight preference for low-frequency (LF) stimuli in terms of perceptibility. However, when asked about comfort and preference, responses shifted toward high-frequency (HF) flickers, indicating that although low frequencies are easier to perceive, high frequencies are generally more comfortable and therefore more suitable for longer-term use.

Regarding authentication methods currently used in daily life, passwords and PIN codes were reported as the most frequently applied, followed by fingerprint and face recognition. This aligns with existing literature on the prevalence of these methods in everyday security practices. Interestingly, TAN codes were reported as rarely or never used, reflecting their decreasing relevance in modern authentication systems. These responses provide an important reference point when comparing the acceptance of EEG-based systems with more established methods.

System usability and workload ratings revealed that participants experienced relatively low mental effort overall, with most ratings clustered toward the lower end of the scale. Eye discomfort was also reported as low by the majority, while tiredness before and after the experiment showed no major increases, suggesting that the experimental paradigm did not induce significant fatigue. Nonetheless, a small number of participants reported higher discomfort or effort, which highlights the importance of minimizing setup complexity and improving electrode comfort in future iterations of the system.

#### 4.4.1 Effect of session time on participant experience

To explore whether the time of day influenced user experience, we compared questionnaire ratings from participants tested before and after 12:00. Eight participants completed the experiment before noon, reporting an average eye strain score of 3.5 on a 6-point scale (1 = no discomfort, 6 = extreme discomfort), whereas those tested in the afternoon reported a slightly higher mean score of 3.92. Similarly, self reported tiredness after the session averaged 4.0 for morning participants and 4.38 on a scale from 1 to 6 (1 = not at all tired, 6 = extremely tired) for afternoon participants. Although these differences are modest, they suggest a mild trend toward greater discomfort and fatigue later in the day, potentially reflecting accumulated daily tiredness on visual sensitivity and cognitive endurance.

## 5 Discussion

The present findings demonstrate that the proposed single-stimulus SSVEP-based authentication system achieves effective performance across a range of experimental conditions, revealing consistent effects of electrode configuration, stimulus frequency, and user-related factors. Several key insights emerge when these results are interpreted in the broader context of BCI research.

First, electrode configuration proved to be a major determinant of performance. The three-electrode setup not only improved accuracy but also stabilized response variability across participants, particularly in the high-frequency condition. This improvement likely stems from enhanced spatial sampling across occipital sites (O1–Oz–O2), which are known to capture the strongest SSVEP responses. In contrast, the single-electrode configuration relies entirely on a single channel's data quality, making it more vulnerable to small changes in impedance or attentional errors.

Filter bank canonical correlation analysis (FBCCA) is a widely adopted, training-free method for steady-state visual evoked potential (SSVEP) recognition and has been shown to achieve high performance particularly in multichannel EEG settings. By decomposing EEG signals into multiple sub-bands and applying canonical correlation analysis across fundamental and harmonic frequencies, FBCCA effectively enhances signal-to-noise ratio and improves frequency discrimination (Chen et al., 2015a). When multiple EEG channels are available, FBCCA can implicitly exploit spatial information by combining signals from different scalp locations, which generally leads to improved robustness and classification accuracy. Consequently, multichannel configurations are often preferred in applications where maximizing decoding performance is the primary objective.

Improving the performance of the established EEG-based BCI system was one of the primary goals of this study. Reducing the number of electrodes was considered a strategic approach to simplify the setup while maintaining accuracy, particularly in the context of authentication systems that prioritize usability and rapid deployment. This aligns with existing literature emphasizing that electrode positions should be functionally relevant to the neural sources being measured rather than arbitrarily dense (Franklin Alex Joseph and Govindaraju, 2021). Although increasing the number of electrodes can further enhance precision by exploiting spatial patterns more fully, it also increases setup time, susceptibility to noise, and computational complexity, thereby reducing suitability for wearable and real-world systems.

Several studies have demonstrated that SSVEP responses are spatially concentrated over the occipital cortex and that a limited set of carefully selected occipital electrodes can preserve most of the task-relevant information. A study by Marx et al. (2019) on SSVEP-based systems showed that over 88.8% of the classification-relevant data originally collected with 16 electrodes could be obtained using only four electrodes (POO1, POO2, O2, and O1). Likewise, Gembler et al. (2017) found that single electrodes placed at Oz achieved the best accuracy, followed by O1 and O2, and that adding additional occipital or parietal electrodes improved results for multi-electrode configurations.

Our findings align with these observations: while single-electrode performance remained competitive, the three-electrode configuration provided a more stable balance between speed, accuracy, and robustness across participants. Informed by these findings, this study employed O1–Oz–O2 as its standard three-electrode configuration. Because these electrodes are adjacent along the occipital line, they are well suited for integration into compact, wearable EEG systems such as headbands. This design choice reflects a forward-looking objective to minimize hardware complexity while maintaining high decoding accuracy, thereby facilitating future translation of SSVEP-based authentication systems into practical, real-world applications.

Second, the results clarify the trade-off between stimulus frequency and user comfort. While low-frequency stimuli traditionally evoke stronger SSVEP responses, they are also more visually intrusive, producing discomfort and potential fatigue over longer trials. In our experiment, high-frequency stimulation (36–45 Hz) offered the best combination of accuracy and user comfort, consistent with earlier findings indicating that higher flicker rates reduce perceptual strain without severely compromising classification reliability. The observation that high-frequency performance remained stable across digits also supports the view that fatigue accumulation was minimal, an encouraging result for real-world, repeated-use authentication tasks.

SSVEP-based visual stimuli, due to their flickering nature, are known to induce visual discomfort and fatigue. This effect is particularly pronounced for low-frequency SSVEP stimuli, which cause greater visual strain than their high-frequency counterparts (Diez et al., 2011). While low-frequency stimuli tend to elicit stronger SSVEP responses (Wang et al., 2006; Ming et al., 2021), they also introduce greater discomfort. In contrast, high-frequency SSVEPs produce less visual fatigue (Diez et al., 2011), making them more suitable for sustained or user-friendly applications.

Although high-frequency paradigms have gained popularity, they still face limitations in classification performance compared to low-frequency systems (Chen et al., 2023). Won et al. (2015) provided direct evidence of this trade-off: participants exposed to both frequency ranges exhibited declining accuracy over time under low-frequency conditions, likely due to fatigue, while high-frequency performance remained stable.

Our findings, on the other hand, suggests high-frequency stimulation appears better suited for long-term comfort and stable performance, but only when paired with multiple electrodes to compensate for its weaker signal amplitude.

Third, the behavioral measures completion time and accuracy indicate that the cognitive demand of the task remained manageable across conditions. The lack of systematic slowdown across digit positions suggests that temporal attention and visual tracking were well maintained throughout the trials. Slightly longer completion times in single-electrode or high-frequency conditions likely reflect reduced SSVEP amplitude rather than increased cognitive load. Importantly, even under these constraints, classification accuracy remained high, highlighting the integrity of the system's feature extraction and decision process.

The shorter completion times observed among female participants may indicate gender-related differences in sustained visual attention or fatigue tolerance. Similar patterns have been hinted at in prior SSVEP-based studies (Volosyak et al., 2011, 2017, 2023), which reported minor yet consistent gender influences on performance stability and comfort. While the current findings are preliminary and drawn from a limited sample, they suggest a trend worth exploring in future work with larger and more balanced participant groups.

From a human factors perspective, the questionnaire responses complement the performance data. Participants generally found the system comfortable and intuitive to use, with minimal reports of eye strain or fatigue. The preference for high-frequency stimuli reinforces the technical findings and underscores the importance of designing BCIs not only for accuracy but also for perceptual comfort and long-term usability. Gender-related trends in completion time, though modest, hint at possible individual differences in visual endurance or flicker sensitivity, suggesting an avenue for more personalized interface design.

### 5.1 Limitations and future research directions

Despite the strong overall performance, several methodological limitations should be acknowledged, alongside opportunities for further development.

First, the experiments were conducted using a high-refresh-rate display (360 Hz), which ensured stable flicker presentation and minimal temporal jitter between visual frames. While this setup provided ideal conditions for precise SSVEP elicitation, such high-end hardware is not typically available to end users. To broaden accessibility, future iterations of the system should be adapted for standard 60 Hz displays. This adaptation will require careful frequency selection and possibly temporal interpolation techniques to maintain stable phase relationships between the visual stimuli and neural responses. Developing a low-refresh-rate variant that remains reliable under common hardware conditions is therefore an essential next step.

Second, occasional frame rate drops were observed during several recording sessions, particularly under high-frequency stimulation. These interruptions likely introduced transient timing inconsistencies that weakened the effective SSVEP signal and may have contributed to the slightly lower accuracy observed in the high-frequency single-electrode condition. Although overall performance remained high, these findings emphasize the sensitivity of high-frequency paradigms to even small deviations in timing. Future implementations should incorporate frame-synchronization monitoring and hardware-level timing validation to ensure consistent stimulus presentation across trials and systems.

Beyond hardware limitations, future work should focus on improving user comfort, adaptability, and consistency across environments. On the hardware side, replacing gel-based electrodes with low-preparation or dry-contact sensors would reduce setup time and enhance usability. Integrating the occipital O1–Oz–O2 positions into a compact headband design with on-board signal conditioning could further improve portability and comfort for everyday applications.

From a signal-processing perspective, user-adaptive and hybrid approaches could enhance decoding stability. Combining SSVEPs with other EEG features such as P300 or error-related potentials may enable attention tracking and automatic correction of misclassifications. Deep learning architectures, including convolutional and spatiotemporal neural networks, also offer promise for faster, more flexible signal interpretation in real time.

Finally, future studies should involve larger and more diverse participant samples and test the system under different lighting and refresh-rate conditions to evaluate generalizability. Extending the paradigm toward continuous authentication or person-specific neural signatures could enable seamless, passive EEG-based identity verification suited for daily use.

### 5.2 Conclusion

This study demonstrated that a single-stimulus SSVEP-based authentication paradigm can achieve high accuracy while maintaining user comfort and simplicity. The results revealed that using three occipital electrodes offers an optimal balance between signal quality and practicality, achieving near-perfect classification accuracy without excessive setup complexity. High-frequency stimuli provided stable and comfortable performance, particularly when sufficient spatial information was available, while low-frequency conditions remained advantageous for faster responses under constrained setups.

Together, these findings illustrate that EEG-based authentication can transition from laboratory-grade systems toward more accessible and wearable designs. By addressing hardware constraints and integrating adaptive signal decoding, future implementations could deliver secure, contactless, and user-friendly brain-computer interface authentication suitable for real-world use.

## Data Availability

The datasets presented in this article are not readily available because access to the data is limited due to data privacy restrictions. All EEG recordings were anonymized and used solely for analysis, and the ethical approval obtained for the study does not permit public sharing of the data.
